# Structural Plasticity Can Produce Metaplasticity

**DOI:** 10.1371/journal.pone.0008062

**Published:** 2009-11-30

**Authors:** Georgios Kalantzis, Harel Z. Shouval

**Affiliations:** Department of Neurobiology and Anatomy, The University of Texas Medical School at Houston, Houston, Texas, United States of America; Center for Genomic Regulation, Spain

## Abstract

**Background:**

Synaptic plasticity underlies many aspect of learning memory and development. The properties of synaptic plasticity can change as a function of previous plasticity and previous activation of synapses, a phenomenon called metaplasticity. Synaptic plasticity not only changes the functional connectivity between neurons but in some cases produces a structural change in synaptic spines; a change thought to form a basis for this observed plasticity. Here we examine to what extent structural plasticity of spines can be a cause for metaplasticity. This study is motivated by the observation that structural changes in spines are likely to affect the calcium dynamics in spines. Since calcium dynamics determine the sign and magnitude of synaptic plasticity, it is likely that structural plasticity will alter the properties of synaptic plasticity.

**Methodology/Principal Findings:**

In this study we address the question how spine geometry and alterations of N-methyl-D-aspartic acid (NMDA) receptors conductance may affect plasticity. Based on a simplified model of the spine in combination with a calcium-dependent plasticity rule, we demonstrated that after the induction phase of plasticity a shift of the long term potentiation (LTP) or long term depression (LTD) threshold takes place. This induces a refractory period for further LTP induction and promotes depotentiation as observed experimentally. That resembles the BCM metaplasticity rule but specific for the individual synapse. In the second phase, alteration of the NMDA response may bring the synapse to a state such that further synaptic weight alterations are feasible. We show that if the enhancement of the NMDA response is proportional to the area of the post synaptic density (PSD) the plasticity curves most likely return to the initial state.

**Conclusions/Significance:**

Using simulations of calcium dynamics in synaptic spines, coupled with a biophysically motivated calcium-dependent plasticity rule, we find under what conditions structural plasticity can form the basis of synapse specific metaplasticity.

## Introduction

Synaptic plasticity is a physiological basis of learning and memory[1]. Experimental studies indicate that influx of calcium into synaptic spines is necessary for induction of bidirectional synaptic plasticity, and that the magnitude and duration of calcium influx determines the sign and magnitude of synaptic plasticity [2], [3]. On the basis of these experimental results, theoretical models of calcium dependent synaptic plasticity (CaDP) have been developed which can account for various forms of induction of both long-term synaptic potentiation (LTP) and long-term depression (LTD) [4], [5], [6]. From a biophysical point of view many parameters influence the time course of synaptically evoked calcium transients in the spine head [7], [8]. Among these, is the geometry of the spine head. Given that calcium transients determine the sign and magnitude of synaptic plasticity, it is reasonable to hypothesize that spine geometry affects synaptic plasticity.

Various experiments have demonstrated morphological changes of dendritic spines that accompany synaptic plasticity, and these changes have been proposed to contribute to alterations in excitatory synaptic transmission during learning [9], [10], [11], [12], [13]. Recent studies suggest that stimulation protocols leading to long-term potentiation (LTP) [14], [15] are associated with increased production of dendritic spines and filopodia and, like LTP itself, this increased spine production is blocked by NMDA receptor antagonist [16], [17], [18]. Using glutamate uncaging, Kasai and collaborators have shown that LTP-inducing stimuli to selected spines in hippocampal pyramidal neurons result in an approximately twofold increase of spine volume [12]. This increase required signaling through N-methyl-D-aspartic acid-type glutamate receptors (NMDAR), calmodulin, and calcium/calmodulin protein kinase II (CAMKII), as well as reorganization of the actin cytoskeleton. In another study of hippocampal pyramidal neurons in acute rat slices, LTD induction was accompanied by decreases in the spine head diameter [19]. Further evidence suggests that there is a link between synaptic potentiation or depression and actin-based spine motility [20], [21]. Fukazawa and colleagues found that dentate gyrus LTP induction is associated with actin cytoskeletal reorganization characterized by a net increase in F-actin content in the dendritic spines[22]. This is consistent with a previous observation that high frequency stimulation (HFS) enlarges the PSD area of polyribosome-containing spines in the hippocampal CA1 region [23]. To summarize, the relationship between structural and functional plasticity looks simple: LTP induces spine enlargement, while LTD induces spine shrinkage. Since LTP and LTD are thought to be essential for memory storage, these results might indicate that spine structure constitutes a structural basis of memory units.

The ability to induce synaptic plasticity does not seem to be constant over time, and it is known that synapse's previous history of activity determines its current plasticity. This activity-dependent modulation of subsequent synaptic plasticity has been termed “metaplasticity”[24]. Candidate mechanism underlying metaplasticity such as changes on the subunit composition of the NMDA receptors, regulation of Group I metabotropic glutamate receptors (mGluRs)[25], endocannabinoid mediated metaplasticity [26] and GABAergic synaptic inhibition [27] have been proposed in the past. This study is based on the observation that alteration of the synaptic weight is accompanied by structural changes of the synaptic spine head. These morphological changes, in turn, result in changes of the calcium dynamics; dynamics that control the induction of synaptic plasticity. We therefore hypothesize that synaptic structural plasticity provides a metaplasticity mechanism for each individual synapse.

In this study we address the question of how spine geometry affects plasticity curves. Based on a simplified compartmental model of the spine head we mimic the morphological changes observed in experimental studies. We use the calcium transients from the compartmental model of the spine, as input for a CaDP model of synaptic plasticity [4], [28]. Using this combined model we simulate two different induction protocols, pairing and spike timing dependent plasticity (STDP). By such simulations we demonstrate how plasticity curves are modified as a function of changes to spine geometry.

Previous models have studies calcium transients during LTP induction protocols, and how they depend on spine geometry[29], [30], [31], [32]. Most studies have used deterministic compartmental models, as we do here, but recent studies have used stochastic simulations [33], [34]. One previous model has suggested that changes in spine geometry can be the basis for metaplasticity, as we suggest here [30]. Our model goes beyond these previous models in that we couple the model of spine diffusion with a synaptic plasticity model in order to more explicitly examine the impact of the structural plasticity of synaptic spines on the induction of subsequent plasticity.

## Materials and Methods

### Compartmental Model of Spine Head

**Figure 1:**
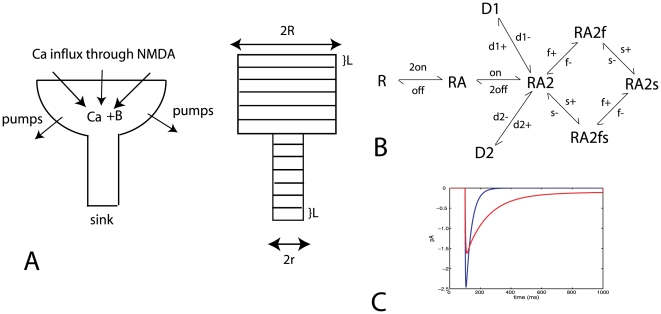
Calcium in the postsynaptic spine. (a) Postsynaptic spine: (Left) Calcium enters the spine head through the NMDA receptors. Ions diffuse inside the spine, react with the calcium buffer B or leave to the extracellular space through the pumps.(Right) Compartmental model of the dendritic spine. R and r are the radius of the head and neck respectively. L is the length of each compartment. (Table 1) (b) Markov model for the NR2A/B subunits of the NMDA receptors (For details see the methods section). (c) Numerical integration of the NMDAR model. The NR2A model (blue) exhibits faster kinetics than the NR2B (red) model. The duration of Glutamate in the cleft is assumed to be 1 ms.

In the past computational studies assume a single compartmental model for the spine head. From our simulations we find that under certain conditions (i.e. geometry of spine neck) there is a spatial variability of the postsynaptic calcium concentration; results that agree with previous studies [34]. Therefore we choose to use a multi-compartmental model for the postsynaptic spine head. The model has 16 compartments (Fig. 1a-right). Six for the spine head and ten for the neck. The end of the spine neck is a trap for the calcium ions. For the *i'th* compartment the dynamics of calcium are described by mass-action kinetics:
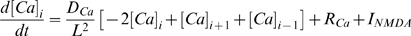
(1)where D_Ca_ is the diffusion coefficient of the calcium ions, L is the length of the *i* compartment. For the coupling of diffusion from the head to the neck, we need to scale the diffusion coefficient with the ratio of their cross sectional areas. In the right hand side of equation 1 the term R_Ca_ represents all the sum of the different calcium sinks. Specifically 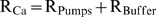. The ATP-driven pumps (R_Pumps_) were modeled with the following equation:
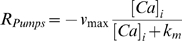
(2)where v_max_ is the velocity of the ATP-driven pump which is equivalent to the product of the pumps density with the membrane area of ith compartment A_i_ divided with the volume of the compartment V_i_. In the dendritic spine there is a large family of calcium binding proteins, among them calmodulin, calbindin and calcineurin. In our model we have used a 2-state Markov model for a generic calcium buffer. The reactions of calcium (R_buffer_) with the buffer can be described by the following two equations:
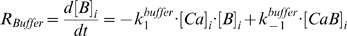
(3)

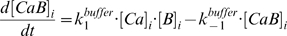
(4)where [B] and [CaB] is the concentrations of free buffer and bound buffer with calcium respectively. The parameters k_1_^buffer^ and k_-1_^buffer^ are the association and dissociation rates.

**Table 1:** Parameters of the compartmental model of the spine head.

Parameter Name	Value
a) Geomery	
L	50 nm
R	200 nm
r	50 nm
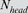	6 compartments
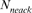	10 copartments
b) reactions	
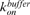	0.5 µM/msec
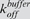	4 µM/msec
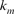	0.5 µM
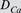	100 nm^2^/msec
G_NMDA_	23.1
[Buffer]_head_	50 µM
[Buffer]_neck_	50 µM
V_max_	3.3
c) NMDA receptors	
N_NMDA_	10
On	31.6/2.83 (mM^.^msec)^−1^
Off	1010/38.1 (msec) ^−1^
d1+	1/550 (10^−3^ mM msec) ^−1^
d2+	1/112 (10^−3^ msec.) ^−1^
d2−	1.01/0.91 (10^−3^ msec.) ^−1^
s+	230/48 (10^−3^ msec) ^−1^
s−	178/230 (10^−3^ msec) ^−1^
f+	3140/2836 (10^−3^ msec) ^−1^
f−	174/174 (10^−3^ msec) ^−1^

When numbers are given as xx/yy the first is for NR2A and the second for NR2B.

The spine head is a complex biological system with various physical compartments and a complex biochemical network. However, by using a simplified model we obtain some intuitive insight about the major “players” of our system. Finally for all the deterministic equations a forward Euler integration method with time step 10^−9^s was sufficient for our model.

### Calcium Influx

In this particular model, calcium enters the spine head from the outermost compartment of the head through the NMDA receptors:
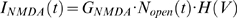
(5)where G_NMDA_ is the conductance of the NMDA receptors, N_open_ is the number of open receptors at time t. The NMDA current is converted to µM of Ca^+2^ by dividing equation 5 with the product 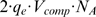, where q_e_ is the charge of the electron, N_A_ is the Avogadro number and V_comp_ is the volume of each compartment of the spine model. We used an 8-states Markov model for the NMDA receptors (Fig. 1b) [35]. The complete set of the kinetic rates for the spine head model are listed in Table 1. The release of Glutamate was modeled as a step pulse of duration 1 ms and amplitude 1 mM. The voltage dependence of the NMDA receptors is described with the following equation [36]:
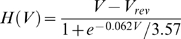
(6)here the reversal potential for calcium is 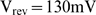. The local depolarization (ie AMPA receptors), is negligible compared with the back propagation action potential (BPAP), therefore we assume 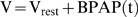.

### Calcium Dependent Plasticity Learning Rule

**Figure 2:**
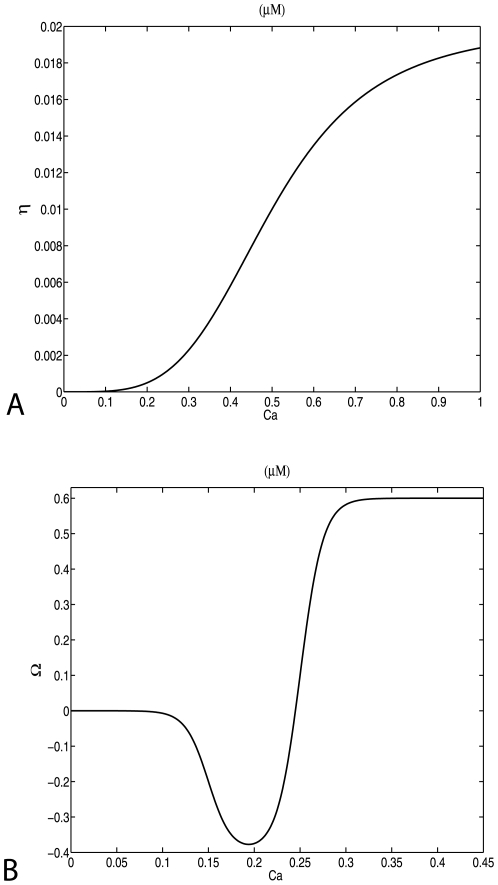
Calcium dependent plasticity model. (a) Learning rate 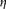 and (b) 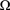 as a function of the calcium concentration.

For modeling plasticity we use the CaDP plasticity model [28]. The model is based on three assumptions. (*i*) that calcium is the primary signal for synaptic plasticity, (*ii*) that the dominant source of calcium influx to the postsynaptic cell is through NMDARs, and (*iii*) that dendritic back-propagating action potentials (BPAPs) contributing to STDP have a slow “after-depolarizing” tail component. The mathematical formulation of the model is the following [28]:
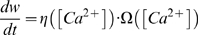
(7)where w is the synaptic weight of the synapse, and [Ca] is the calcium concentration at that synapse and the Ω function, as depicted in figure 2b, has the form:
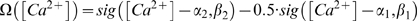
(8)where sig(x,β) = exp(βx)/(1+exp(βx)) and α_0_ = 0.4, α_ 1_ = 0.150, α_ 2_ = 0.250, β_1_ = 80, β_ 2_ = 80. The calcium dependent learning rate function, η, defines how fast the synaptic weights change each time that we have synaptic activity (Fig. 2a) and has the form:
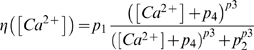
(9)where p_1_ = 0.02, p_2_ = 0.5, p_3_ = 4.0 and p_4_ = 10^−7^. This learning rate function has a sigmoidal form, which monotonically increases with Ca^2+^. The general form of equations 7 and 8, has been derived from lower level biophysical models [37], [38]. The form of the Ω function is based qualitatively on the notion that a moderate rise in calcium produces LTD whereas a large rise in calcium produces LTP. The actual parameters were set arbitrarily in order to obtain reasonable plasticity curves, given the calcium transients assumed here.

For the BPAP we used a double exponential function:
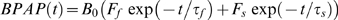
(10)

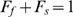
(11)where B_0_ ( = 10 mV) is a constant, F_f_ ( = 0.7) and F_s_ ( = 0.3) is the relative amplitude of the fast and slow component of the BPAP whereas 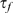
( = 2 ms) and 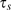 ( = 30 ms) are the time constants of the fast and slow component respectively.

### Plasticity Protocols

The induction of long-term potentiation (LTP) in the hippocampal CA1 region requires both presynaptic activity and large postsynaptic depolarization. A standard protocol for inducing LTP using whole-cell recording is to pair low-frequency synaptic stimulation (100–200 pulses, 1–2 Hz) with a depolarizing voltage-clamp pulse (1–3 min duration). During that pairing protocol, when we vary the postsynaptic voltage we achieve induction of potentiation or depression. Specifically, for lower values of the voltage long-term depression (LTP) is induced while for higher values we have induction of long-term potnetiation (LTP) is induced. However, the magnitude and direction of synaptic plasticity can be determined by the precise timing of presynaptic and postsynaptic action potentials on a millisecond timescale. With the STDP protocol repeated presynaptic and postsynaptic stimulation, separated by a fixed interval (Δt) is applied. If the presynaptic spike arrives a few milliseconds before the postsynaptic action potentials then we have induction of LTP whereas if the presynaptic action potential arrives after the post we have LTD. A postive sign of the time interval Δt implies a pre-post condition where as a negative sign a post-pre.

This plasticity model predicts two LTD windows, the standard window when the presynaptic spike comes after the postsynaptic (Δt<0) spike, and an additional LTD window at Δt>0, but at larger values than the LTP window. This second LTD window is consistent with STDP induced in Hippocampal slices [39], [40]. However, it might not be consistent with STDP in other systems. We have previously shown that stochastic synaptic transmission can significantly reduce the magnitude of this second window, even when using the same learning rule [41].

### Changes in Spine Head Volume and NMDA Receptor Conductance

Two alternatives as to how the NMDA receptor conductances are changed subsequent to changes in spine volume are considered. One alternative assumes that the changes in conductance are proportional to the changes in the volume and the other alternative assumes changes proportional to the surface area of the spine. What is actually different in the two protocols is the way in which way the volume is changed. However, these different ways of changing the volume naturally lead to a different relative compensation of NMDAR conductance the volume altered spine.

#### 1. Changes proportional to volume

This is implemented by changing the volume (V) only through changes in the spine radius (R'), and changing the NMDA receptors current (I_NMDA_) proportionally to this parameter as well. Mathematically this can be formulated:
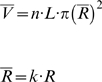
(12)where, R is the initial spine hear radius, l the spine length and k a scaling factor controlling the change in volume.

Here we change the NMDA current according to:
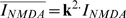
(13)


#### 2. Changes proportional to surface area

This is implemented by changing the volume of the spine proportionally in all dimensions, but changing the NMDA receptor conductance proportionally to the cross sectional area of the spine (πR^2^). That reflects the case where the number of the NMDA receptors is a function of the cross sectional area of the spine head, or similarly proportional to the area of the postsynaptic density. Therefore, here:
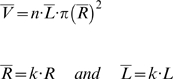
(14)while
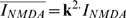
(15)


## Results

### Morphological Changes of Dendritic Spine Head Modify Plasticity Curves

**Figure 3:**
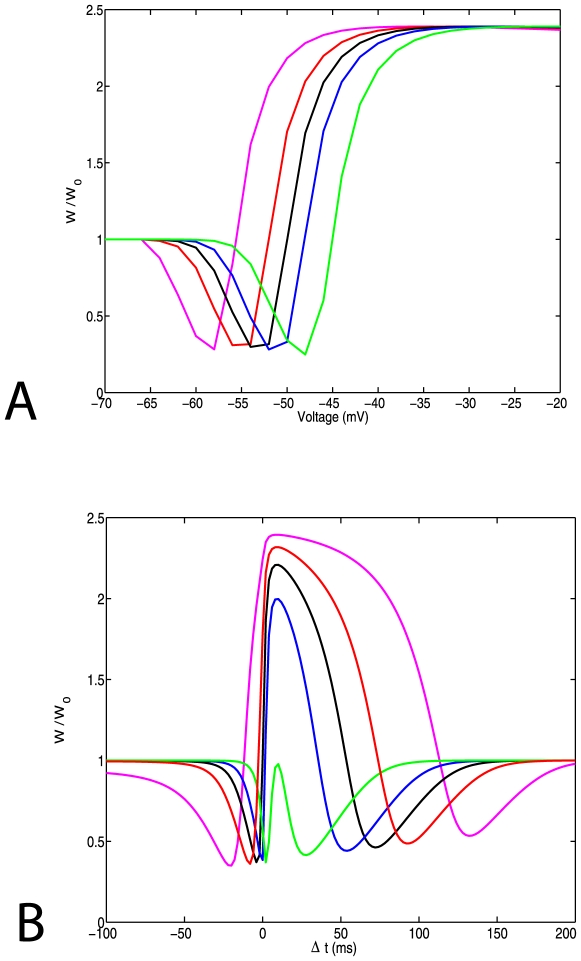
The effect of spine geometry on synaptic plasticity. (a) Plasticity curves for a pairing protocol. (b) Plasticity curves for a STDP protocol. Here we use NR2B receptor dynamics. The radius of spine head: 160,185,200,215,240 (magenta, red, black, blue, green) is altered in different simulations, affecting the resulting plasticity curves.

Experimental evidence suggests that during LTP or LTD the volume of the synaptic spine head changes. Based on a simplified compartmental model of the spine head we studied alterations of the plasticity curves as a function of the spine volume for two protocols for induction of plasticity. Using a cylindrical compartmental model (Fig. 1a) there are three different ways of changing the spine head volume: 1. Increasing the radius R of each compartment. 2. Increasing the length L of each compartment. 3. Changing both the radius and the length. First, we change the radius and we keep constant the number of NMDA receptors and the concentration of the buffer. Figure 3 illustrates plasticity curves for the pairing (3a) and STDP induction protocols (3b). Both curves use NR2B kinetic rates.

The STDP plots exhibit a relatively wide LTP window compared with experimental results [39]. The widths of the different plasticity windows arise from the different model parameters, and in particular from the NMDA receptor time constant [4], [42]. Here we used a slow NMDAR receptor consistent with the NR2B receptor subtypes, if a shorter NMDAR time constant, consistent with NR2A receptors is used (Figure S1), a shorter LTP window is obtained. However, these precise details do not affect the main qualitative point being made here.

Our results indicate that the threshold for LTP increases as we increase the volume of the spine head. This happens because the volume of the spine increases, but the magnitude of calcium influx does not, resulting in a dilution of the calcium ions in the spine. Hence, as a result of increases in the spine volume further increase of the synaptic weight becomes more difficult. Modifying the spine volume by changing the length of each compartment or by changing simultaneously the radius and the length produces similar results. These results were obtained with kinetic coefficients appropriate for NR2B receptors. By using appropriate kinetic rates for NR2A receptors qualitatively similar results are obtained, although the curves are shifted, as previously demonstrated [37].

### Alterations of NMDA Current Brings Plasticity Curves to the Initial State

Alterations of the synaptic weight are due to the exocytosis (or endocytosis) and phosporylation of AMPA receptors at the postsynaptic spine. One study observed that the rapid increase in the number of NMDA receptors after the induction of LTP is followed by a delayed though proportional increase of the NMDA current [43]. The effect of the delayed potentiation of the NMDA currents on the plasticity curves can be simulated in our model. We tested two different scenarios for the increase of the NMDA current. First, we assumed that the number of the NMDA receptors increases proportionally to the total spine volume. This is implemented by changing the volume (V) only through changes in the spine radius (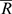), and changing the NMDA receptors current (I_NMDA_) proportionally to the surface area of the spine head (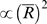), as described mathematically by equations 11,12 in the methods section.

**Figure 4:**
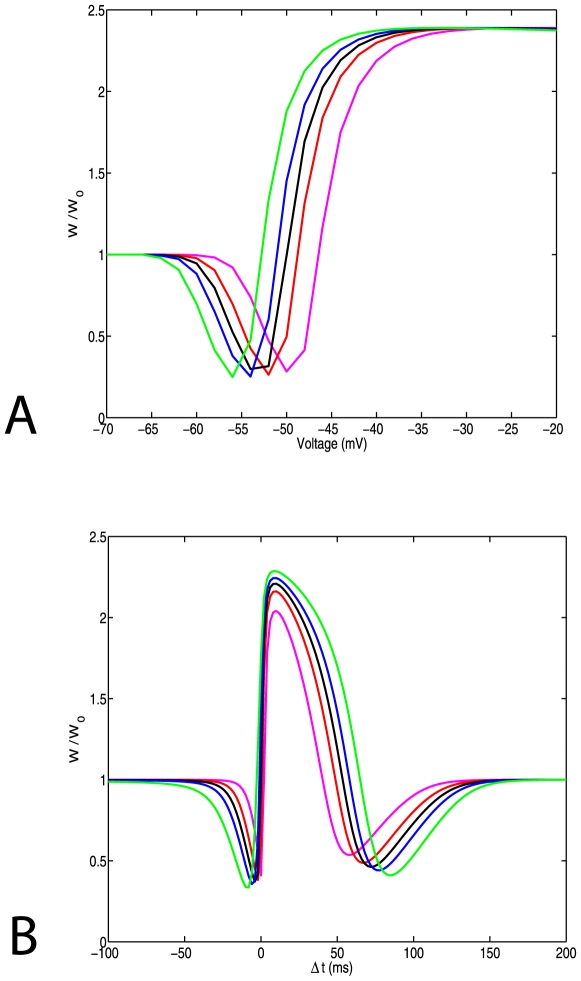
The effect of changing NMDA receptor currents proportionally to spine volume. (a) Pairing protocol. (b) STDP protocol. Both protocols were induced using an NR2B kinetic model. Conductance of NMDA as a function of the Radius: 160,185,200,215,240 (magenta, red, black, blue, green). Here the relation between head radius and LTP threshold is reversed when compared to figure 3.

Plasticity curves, after implementing this change of spine volume and NMDA receptor conductance, are shown in Fig. 4. After the NMDA receptor conductance is modified, the threshold for LTP is smaller for the larger spine. Therefore the plasticity curves have been reversed (Compare to Fig. 3). This arises because of the increase in calcium influx in the larger spines and its decrease in the smaller spines.

In the second scenario, we assumed that the number of NMDA receptors is proportional to the surface of the spine head. This is implemented by changing the volume of the spine proportionally in all dimensions, but changing the NMDA receptor conductance proportionally to the cross sectional area of the spine (πR^2^). That reflects the case where the number of the NMDA receptors is a function of the cross sectional area of the spine head, or similarly proportional to the area of the postsynaptic density. This is described mathematically by equations 13,14.

**Figure 5:**
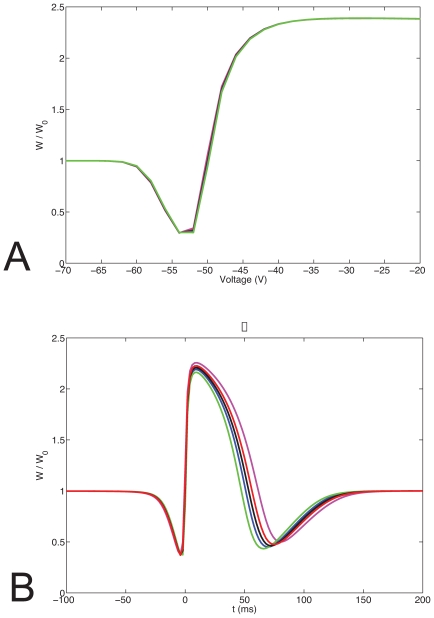
The effect of changing NMDA receptor currents proportionally to the surface area of the spine. (a) Pairing protocol and (b) STDP protocol. Conductance of NMDA receptors is set to be proportional to the surface area of the spine head **(πR^2^)**. Spine volume is changed scaling the radius linearly with L. In that way the conductance of the NMDA receptors is proportional to R^2^ where as the volume to R^3^. The basal values of radius and length are: R = 200 nm, spine head length = 300 nm (L = 50 nm, N_Head_ = 6). Scaling factors [0.86177 0.94935 1.0 1.049395 1.1292447] (magenta, red, black, blue, green).

Under these conditions (Fig. 5) the plasticity curves for both the pairing and the STDP protocols are nearly identical to those observed before the volume increase (Fig 3, black line), and are independent of the spine volume (initial radius of spine head 200 nm). Therefore when the number of the NMDA receptors are scaled proportionally with the cross sectional area, the plasticity curves return back to their initial state.

## Discussion

Experimental evidence suggests that protocols that cause changes in synaptic weights also cause changes of spine geometry [9], [10], [11], [12], [13]. It is well established that calcium transients in spines are required for induction of synaptic plasticity, and that the sign and magnitude of synaptic plasticity is influenced by the magnitude and duration of these calcium transients [2], [3]. Biophysically, synaptic calcium transients are dependent on the morphology of the dendritic spine head, and therefore activity dependent changes of synaptic morphology are also likely to affect synaptic plasticity. Therefore, a link between induction of plasticity and synaptic geometry may exist. In this paper we address the question how spine geometry may affect plasticity.

In this study we use a simplified biophysical compartmental model of the spine head for simulating calcium dynamics. The source of calcium influx in our spine model is through the NMDA receptors, and calcium is removed by calcium pumps, binding to a calcium buffer, and diffusion through the spine neck. The parameters for the geometry of this model are based on anatomical measurements [44], [45]. In combination with the CaDP plasticity model we simulated induction of plasticity for two different protocols, pairing and STDP.

First, we simulated the effect that changes in spine geometry have on the induction of synaptic plasticity. We assumed that the only changes are to the spine geometry and that there are no changes in the number of NMDA receptors or their properties. We demonstrated that modifications in the spine geometry produce a change in the plasticity curves and a shift of the threshold between LTP and LTD. An increase in spine volume causes an increase in the LTD/LTP threshold. Consequently the induction of LTP, which is accompanied by an increase in spine volume, makes the synapses less liable for additional LTP. Our results resembles the sliding threshold of the BCM [46] model, and demonstrates that structural plasticity may provide a negative feedback loop to maintain synaptic strength and plasticity within a functional dynamic range. These results also differ from BCM, because the negative feedback due to structural plasticity is synapse specific, unlike the whole cell-sliding threshold postulated by BCM. Such a possibility has been previously discussed [30], but here by combining a spine model with a plasticity model, we show that the modification threshold can indeed shift.

Many experimental results show that synaptic plasticity causes changes in the AMPA receptor currents that are not accompanied by equivalent changes to NMDA receptor dependent currents. However, a paper by Watt et al. (2004) shows that LTP initially causes an increase only of the AMPA current, but this is followed later by a proportional increase in the NMDA receptor current. In order to test how that increase of the NMDA current may affect plasticity we considered two different assumptions. First, we assumed that this increase of the NMDA current is proportional to the volume of the spine head. Our simulation results show that for both induction protocols the plasticity curves have been reversed (Fig. 4). In pairing protocols the threshold of LTP/LTD is now smaller for larger spines, and STDP protocols now have wider temporal windows in the larger spines. These results indicate a positive rather than a negative feedback mechanism, and are unlikely to stabilize plasticity. Second, we assumed that the increase of the NMDA current is proportional to the surface area of the spine head, which is likely proportional to the area of the PSD. This is a reasonable assumption because it is likely that the number of membrane bound receptors will become proportional to the area of the membrane. When the number of NMDA receptors is proportional to the surface area the plasticity curves are returned to their initial state (Fig. 5). In other words, larger and smaller synapses have approximately the same potency for learning. This delayed increase in the number of NMDA receptors has the effect of setting up a refractory period for plasticity.

One might expect that changing the NMDA receptor conductance proportionally to the spine volume would compensate for the dilution of the calcium due to the increase of the spine head volume, and therefore the plasticity curves will return to their original shape. Instead we find that changing conductance proportional to the volume over compensates for the morphological changes. This might seem surprising, however when a spine gets larger, the size of the spine neck is not changed, and therefore the relative sink due to diffusion across the neck is smaller. Similarly, when a spine gets smaller with a fixed neck size the relative sink gets larger. For this reason a rescaling of NMDAR conductance proportional to the volume over compensates. We also find that a rescaling of NMDAR conductance proportional to the change in the spine surface area, which seems biologically more plausible, does approximately return the plasticity curves to their form before the morphological changes. These results could not be obtained with a single compartment model of a spine, unless the sink and source coefficients were modified appropriately for each different spine volume, in order to emulate the results that naturally arise from the multi-compartment model.

The plasticity model we have used is simplified compared to the biological mechanisms operating in the brain. It does not take into account non-NMDAR sources of calcium, and plasticity that is NMDA receptor dependent. It produces two LTD windows, consistent with some experimental results [39], [40] but not others. We have previously shown that including the effects of stochastic synaptic transmission can significantly affect the magnitude of the second LTD window [41]. However, despite of the limitations of this simple model, we were able to demonstrate the effect of structural plasticity on subsequent synaptic plasticity. We expect that the consequences of spine volume change, and subsequent changes of NMDAR conductance would generalize to more complex models as well.

Experimental evidence suggests rapid changes in spine morphology, on a time scale of minutes. Those include fluctuations and fast shrinkage in response to glutamate [10], [47] as well as expansion or shrinkage depending on the intensity of the stimulation [48]. However, in organotypic hippocampal slices, electron microscope (EM) 3D reconstruction, revealed no changes of the overall spine size 2 hours post-tetanus [49]. Similarly, some in vivo studies suggested no net change in the average volume or diameter of the spines from young or adult mice [50], [51]. However, EM studies show a positive correlation between spine head size, PSD area, and AMPAR immunolabeling [44], [52], suggesting that plasticity indeed is correlated with changes in spine volume. Recent physiological evidence [53] demonstrated that repetitive pairing of postsynaptic spikes and two-photon uncaging of glutamate at single spines produced two distinct phases of spine enlargement in CA1 pyramidal neurons. The first phase exhibited a rapid (15–20 min) increase of volume that is not protein synthesis dependent. The second phase is a gradual and persistent increase of spine volume that is protein synthesis dependent. Combining these results with other results about protein synthesis dependent plasticity [54], indicates that the induction of plasticity produces transient morphological changes of the spine head accompanied with exocytosis of AMPA receptors. At the second phase, new protein synthesis adds postsynaptic density proteins to stabilize these receptors in the membrane and consequently the shape of the synapse, and possibly more NMDA receptors are added. In addition the results of Watt et. al (2004) indicate that the early increase of spine volume and AMPA receptor currents in followed (40–50 min) by a proportional increase in NMDA receptor currents.

Based on a simplified model of the spine we demonstrated that after the induction phase of plasticity a shift of the LTP/LTD threshold takes place. This induces a refractory period for further LTP induction and promotes depotentiation as observed experimentally. In the second phase, alteration of the NMDA response may bring the synapse to a state such that further synaptic weight alterations are feasible. We showed that if the enhancement of the NMDA response is proportional with the area of the PSD the plasticity curves most likely return to the initial state.

## Supporting Information

Figure S1 Plasticity curves for two plasticity protocols (pairing and STDP) when kinetics of NR2A subunits is used. We notice the reduced LTP window compared with that of NR2B.
(1.25 MB EPS)
